# Emergence of De Novo Conditions Following Remission of Cushing Syndrome: A Case Report and Scoping Review

**DOI:** 10.1002/edm2.476

**Published:** 2024-04-10

**Authors:** Noémie Desgagnés, Laura Senior, Daniel Vis, Katayoun Alikhani, Kirstie Lithgow

**Affiliations:** ^1^ Department of Medicine University of Calgary Calgary Alberta Canada; ^2^ Division of Respirology, Department of Medicine University of Calgary Calgary Alberta Canada; ^3^ Department of Clinical Neurosciences University of Calgary Calgary Alberta Canada; ^4^ Division of Endocrinology, Department of Medicine University of Calgary Calgary Alberta Canada

**Keywords:** adult, autoimmune diseases, Cushing syndrome, glucocorticoids, humans, sarcoidosis, thyroiditis

## Abstract

**Objective:**

Onset and exacerbation of autoimmune, inflammatory or steroid‐responsive conditions have been reported following the remission of Cushing syndrome, leading to challenges in distinguishing a new condition versus expected symptomatology following remission. We describe a case of a 42‐year‐old man presenting with new‐onset sarcoidosis diagnosed 12 months following the surgical cure of Cushing syndrome and synthesise existing literature reporting on de novo conditions presenting after Cushing syndrome remission.

**Methods:**

A scoping review was conducted in Medline, Epub, Ovid and PubMed. Case reports and case series detailing adult patients presenting with new‐onset conditions following Cushing syndrome remission were included.

**Results:**

In total, 1641 articles were screened, 138 full‐text studies were assessed for eligibility, and 43 studies were included, of which 84 cases (including our case) were identified. Most patients were female (85.7%), and the median reported age was 39.5 years old (IQR = 13). Thyroid diseases were the most commonly reported conditions (48.8%), followed by sarcoidosis (15.5%). Psoriasis, lymphocytic hypophysitis, idiopathic intracranial hypertension, multiple sclerosis, rheumatoid arthritis, lupus and seronegative arthritis were reported in more than one case. The median duration between Cushing remission and de novo condition diagnosis was 4.1 months (IQR = 3.75). Of those patients, 59.5% were receiving corticosteroid therapy at the time of onset.

**Conclusion:**

Our scoping review identified several cases of de novo conditions emerging following the remission of Cushing syndrome. They occurred mostly in women and within the year following remission. Clinicians should remain aware that new symptoms, particularly in the first year following the treatment of Cushing syndrome, may be manifestations of a wide range of conditions aside from adrenal insufficiency or glucocorticoid withdrawal syndrome.

## Graphical abstract

Onset and exacerbation of autoimmune, inflammatory or steroid‐responsive conditions have been reported following the remission of Cushing syndrome, leading to challenges in distinguishing a new condition versus expected symptomatology following remission. We describe a case of a 42‐year‐old man presenting with new‐onset sarcoidosis diagnosed 12 months following the surgical cure of Cushing syndrome and synthesise the existing literature reporting on de novo conditions presenting after Cushing syndrome remission. Our scoping review identified several cases de novo conditions emerging following the remission of Cushing syndrome occurring mostly in women and within the year following remission.

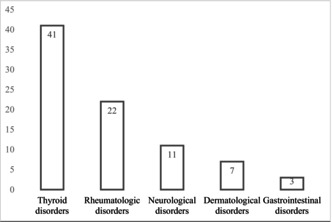

## 1 Introduction

Cushing syndrome (CS) is caused by chronic exposure to excessive glucocorticoids, from either endogenous or exogenous sources [1]. Endogenous Cushing syndrome can be classified as either adrenocorticotropic hormone (ACTH) dependent or independent. ACTH‐dependent causes comprise 80% of cases, most of which are pituitary corticotroph adenomas. Unilateral adrenal adenomas are the most common ACTH‐independent cause, comprising 20% of total cases [2]. Treatment focuses on controlling tissue exposure to cortisol and treating the source of cortisol overproduction, which can be achieved through surgical resection, radiation or medical therapy depending on the underlying aetiology [2].

Following the biochemical remission of Cushing syndrome, patients commonly feel unwell due to adrenal insufficiency (AI) and/or glucocorticoid withdrawal syndrome (GWS). AI is an expected consequence of remission due to the chronic suppression of the hypothalamic‐pituitary‐adrenal (HPA) axis from glucocorticoid excess and can manifest with heterogeneous symptoms including myalgias, muscle weakness, fatigue, hypersomnolence, anorexia, nausea and abdominal discomfort [3, 4, 5]. GWS is due to the dependence on supraphysiologic glucocorticoid levels and has overlapping symptoms with AI, but occurs even with physiologic or supraphysiologic glucocorticoid replacement [5]. Both AI and GWS can persist for 1 year or longer following the remission of Cushing syndrome [5].

Due to immunosuppression induced by glucocorticoid excess [1, 6, 7], the remission of Cushing syndrome has the potential to unmask or aggravate an underlying autoimmune, inflammatory or steroid‐responsive condition. Reports of such conditions include thyroiditis, psoriasis, sarcoidosis and systemic lupus erythematosus (SLE) [8, 9, 10, 11]. Therefore, persisting symptoms following the remission of Cushing syndrome can be due to AI, GWS or presentation of a new condition. The latter situation may evade timely diagnosis since AI and GWS are expected consequences in this clinical setting.

We report a case of a 42‐year‐old patient with Cushing syndrome secondary to an adrenal adenoma with first presentation of sarcoidosis 12 months after adrenalectomy. We performed a scoping review to synthesise previous reports of de novo autoimmune, inflammatory or steroid‐responsive conditions following the remission of Cushing syndrome. Our aim was to characterise these presentations to provide guidance to clinicians in making this diagnosis challenging.

## 2 Case Report

A 42‐year‐old white man was referred to endocrinology with a 1‐year history of insomnia and rapid weight gain of 18 kg. Past medical history was significant for a pituitary lesion presumed to be a Rathke's cleft cyst, which had been stable on neuroimaging for over two decades. He was otherwise healthy with no prescribed medications. On physical examination, blood pressure was 159/99 mmHg. Pertinent findings included facial plethora, dorsal and supraclavicular fat pads, reduced skin thickness and multiple violaceous striae on the abdomen. Biochemistry showed elevated 24‐h urine cortisol on two occasions (3067.5 nmol/day, 2704.0 nmol/day; reference range, 100.0–380.0 nmol/day) and elevated late‐night salivary cortisol (54.2 nmol/L; reference range, ≤ 3.6 nmol/L). Plasma ACTH level was suppressed (< 1.1 pmol/L; reference range, 2.0–11.5 pmol/L). Serum‐free thyroxine (fT4), thyroid‐stimulating hormone (TSH), follicle‐stimulating hormone (FSH), luteinising hormone (LH) and free testosterone were all within normal limits. Serum random glucose level was normal (4.6 mmol/L; reference range, 3.3–11.0 mmol/L), and haemoglobin A1c (HbA1c) was within the pre‐diabetes range at 6.2% (6.0%–6.4%). His serum complete blood count, sodium, potassium and creatinine levels were all within normal limits. His body surface area was 2.53 m^2^.

The patient was diagnosed with ACTH‐independent Cushing syndrome. Computed tomography of the abdomen and pelvis revealed a 4.8‐cm mass in the left adrenal gland. The patient was referred to endocrine surgery, and in the interim, medical treatment with ketoconazole 200 mg p.o. twice daily and spironolactone 50 mg p.o. daily was initiated, which resulted in normalisation of his 24‐h urine cortisol. Shortly after initiating these medications, the patient noticed paraesthesia in his extremities. There was no objective evidence of neuropathy on physical examination, and laboratory investigations including vitamin B12 (329 pmol/L; reference range, 155–700 pmol/L), TSH (2.14 mIU/L) and follow‐up HbA1c (5.7%) were within normal range.

Three months following his initial presentation, the patient underwent left adrenalectomy. Postoperatively, supraphysiologic glucocorticoids were initiated and he was discharged home on oral hydrocortisone 40 mg in the morning and 20 mg in the afternoon. Pathology was consistent with an adrenal cortical adenoma with Ki‐67 < 1%.

The patient was highly motivated to wean his glucocorticoid doses to ameliorate symptoms of cortisol excess. He tapered his hydrocortisone to 20 mg in the morning and 10 mg in the late afternoon within 2 weeks postoperatively. He developed significant muscle stiffness to his shoulders, with diffuse myalgias and arthralgias, along with worsening of his pre‐existing paraesthesia. Four months after the surgery, he had further reduced his hydrocortisone to 15 mg in the morning and 5 mg in the late afternoon with improvement in his Cushingoid features (reduced supraclavicular fullness, reduced abdominal adiposity, fading of abdominal striae and seven‐kilogram weight loss). He was assessed by neurology for his paraesthesia, but no organic cause was identified.

**FIGURE 1:**
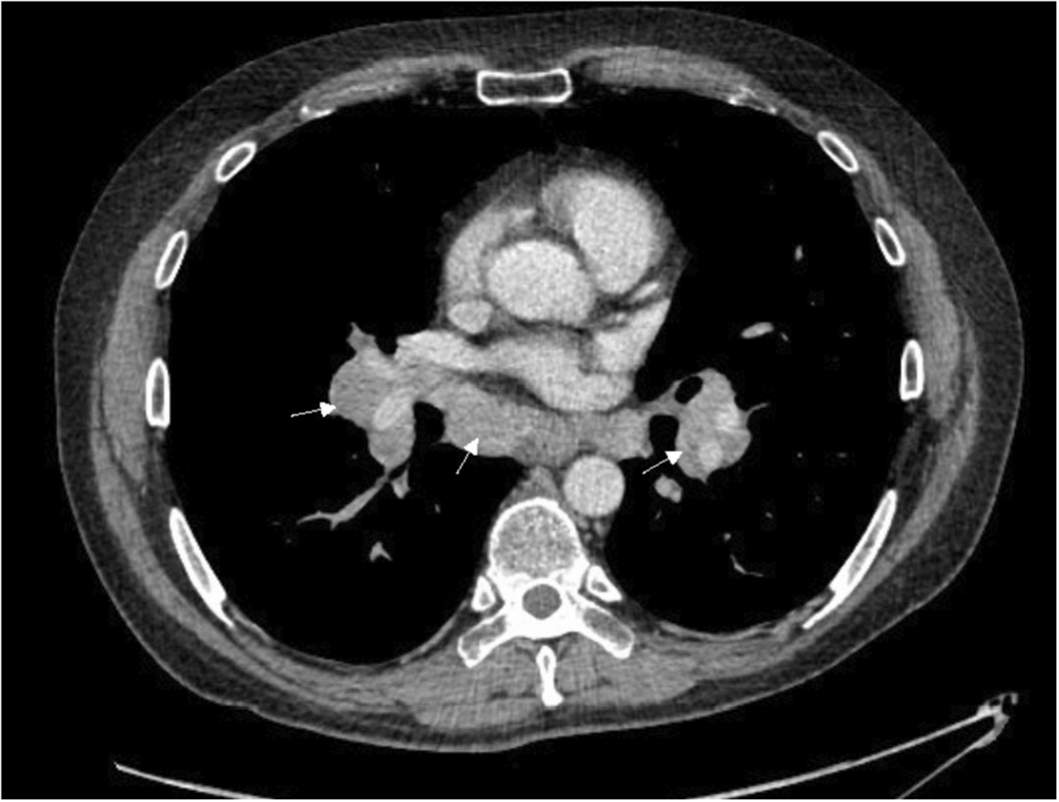
Enhanced CT scan of the chest demonstrating bilateral hilar and mediastinal lymphadenopathy (indicated by arrows).

Twelve months after surgery, he had weaned off his hydrocortisone to 5 mg twice daily and continued to feel unwell with headaches, muscle weakness and morning stiffness. Morning cortisol after withholding glucocorticoids for 24 h was 35 nmol/L (170–500 nmol/L), demonstrating ongoing HPA axis suppression. The patient's family physician ordered a chest X‐ray for a prominent sternoclavicular joint, and the patient was incidentally found to have bilateral hilar lymphadenopathy. The patient was referred to respirology and underwent bronchoscopic sampling of his mediastinal lymph nodes (see Figure 1), which demonstrated well‐formed non‐necrotising granulomas from lymph node Stations 7 and 11L. Cultures for fungi, AFB and flow cytometry were all negative, confirming Stage 2 pulmonary sarcoidosis. There was no indication for sarcoidosis‐specific treatment with glucocorticoids, cytotoxic agents or biologics based on his normal pulmonary function testing and lack of active extra‐pulmonary sarcoidosis. However, given the ongoing HPA axis suppression, hydrocortisone was empirically increased to 20 mg total daily dose, which led to improvement in the patient's symptoms.

Due to the ongoing symptoms of headaches and known pituitary lesion potentially concerning for neurosarcoidosis, the patient was referred to neuroimmunology. MRI brain, and cervical, thoracic and lumbar spine showed a reduction in the size of the known cystic pituitary lesion, with no findings suggestive of intracranial or spinal sarcoidosis, and no abnormal leptomeningeal enhancement. Electromyography demonstrated normal nerve conduction studies.

Two years following adrenalectomy, the patient has weaned off all glucocorticoid replacement with resolution of his symptoms of adrenal insufficiency. His sarcoidosis remains in remission.

## 3 Methods

A scoping review protocol was developed using the Joanna Briggs Institute methodology [12]. We followed the Preferred Reporting Items for Systematic Reviews and Meta‐Analyses (PRISMA) extension for Scoping Reviews guidelines in reporting our protocol and results [13].

### 3.1 Systematic Literature Search

A preliminary search strategy was developed with the aid of a medical librarian. The full search strategy and terms are presented in Appendix 1. Ovid MEDLINE and Epub Ahead of Print, In‐Process, In‐Data‐Review & Other Non‐Indexed Citations and Daily and PubMed databases were searched from inception to 8 September 2022. Additional articles were identified by searching the reference lists of all included articles.

### 3.2 Eligibility Criteria

We considered descriptive observational studies including case series and case reviews, as well as systematic reviews. Articles from all years and locations were included; however, articles written in another language than in English or French were excluded given the limitations in conducting review and data extraction from these sources. Full inclusion and exclusion criteria are shown in Table 1. We included the reports of adults ≥ 18 years of age with endogenous Cushing syndrome with a de novo presentation of an autoimmune, inflammatory or steroid‐responsive condition following remission, which could be induced by surgery, radiotherapy, medical therapy or a combination of these treatments. Cases of Cushing syndrome secondary to exogenous corticosteroids were excluded due to the high likelihood of pre‐existing steroid‐responsive conditions in this population. Flares or recurrences of previously diagnosed inflammatory, autoimmune or steroid‐responsive conditions were also excluded. Patients with Cushing syndrome secondary to metastatic cancer (i.e. metastatic corticotroph adenoma or metastatic adrenocortical carcinoma) were excluded. Remission was defined as clinical and/or biochemical evidence of AI following treatment of CS by any modality.

**TABLE 1:** Scoping review inclusion and exclusion criteria.

Inclusion criteria	Exclusion criteria
Studies published in any year and location	
Studies published in English and French	Studies published in another language than in English or French
All adults ≥ 18 years old at the time of Cushing syndrome cure	Children < 18 years old
Endogenous Cushing syndrome	Exogenous Cushing syndrome
De novo conditions post‐remission	Pre‐existing conditions with flare post‐remission
	Cushing syndrome caused by metastatic cancer

### 3.3 Study Selection

All identified studies were uploaded to Covidence, and duplicate articles were removed. Titles and abstracts were screened for eligibility by one reviewer, and articles without abstracts were screened in totality for eligibility. Selected articles underwent a full‐text review by two reviewers for inclusion. Disagreements about eligibility of an article were resolved by a third reviewer.

### 3.4 Data Extraction

Two members of the study team created a data extraction tool to collect patient characteristics from the studies that met eligibility criteria following a full‐text review. The data extraction tool was piloted with all study team members, and adjustments were made as needed. Patients' age, gender, aetiology of Cushing syndrome, treatment modality and de novo condition were recorded. Characteristics of de novo conditions were collected including clinical presentation, timing of onset, presence of exogenous steroids at the time of presentation and resolution. Data from all included studies were extracted independently by two study team members and reconciled. Any discrepancies were resolved by referring to the primary article.

### 3.5 Statistical Analysis

In this descriptive study, categorical variables are expressed as percentages and non‐normally distributed continuous variables as median and interquartile range (IQR). Median and IQR were preferred over mean and standard deviation given the small sample size.

## 4 Results

**FIGURE 2:**
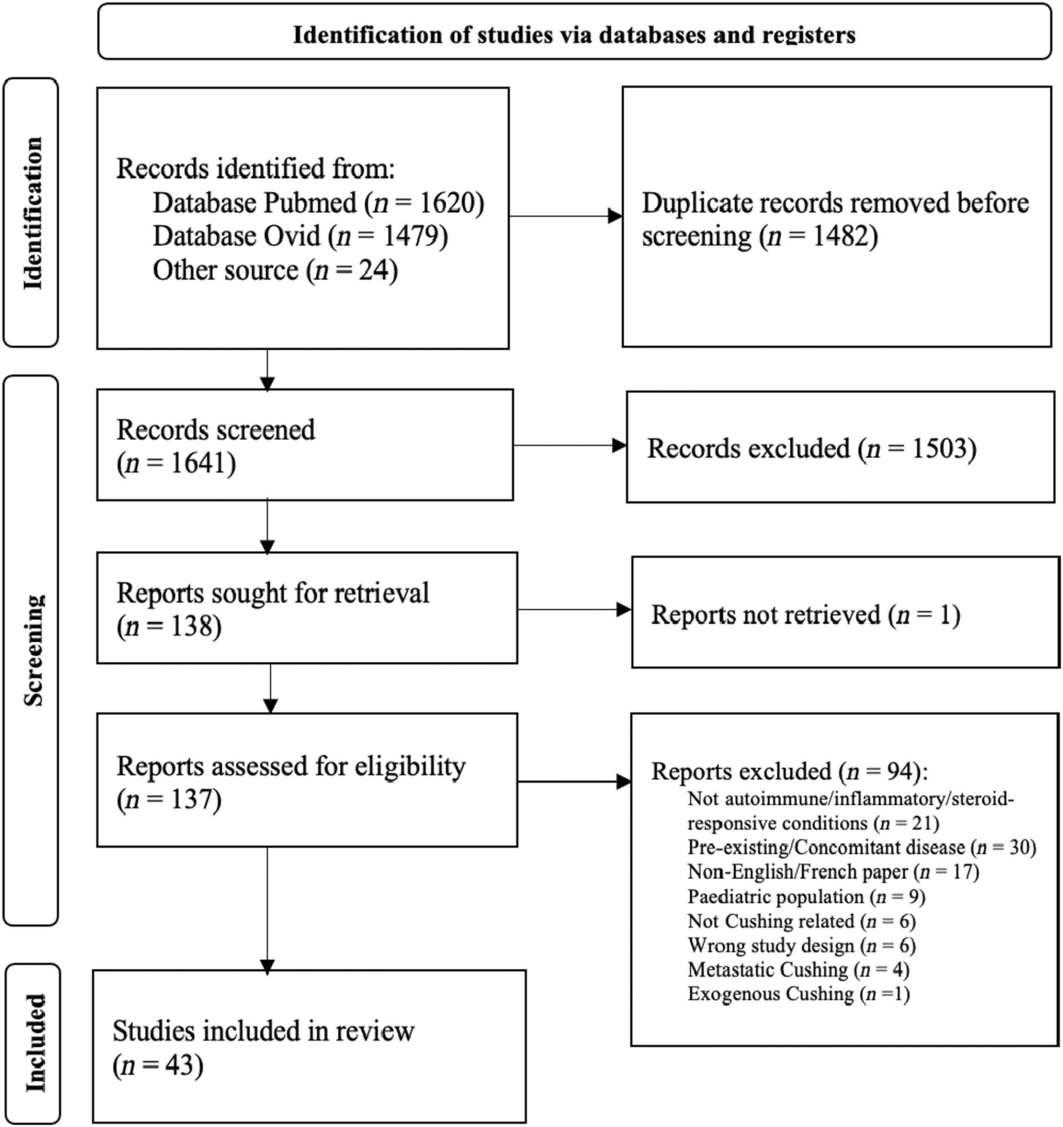
PRISMA flow diagram of included studies.

The search strategy identified 3123 total citations: 3099 abstracts from database searching and 24 from hand‐searching (Figure 2). There were 1641 citations remaining after duplicates were removed. After title and abstract screening, 138 studies underwent full‐text review, and 43 studies were included in data extraction and analysis (see Appendix 1 for a full list of included citations).

All included studies were either case reports (*n* = 34) or case series (*n* = 9). Five articles [8, 9, 14, 15, 16] also included a literature review and four [8, 10, 11, 17] included cohort studies in addition to the case report/series. Included articles were published from 1981 to 2021 inclusively. These 43 studies identified 83 unique patient cases of new‐onset conditions following the remission of Cushing syndrome (see Table 2 for full patient characteristics). In addition to our case, this review includes 84 cases. Most patients were female (*n* = 72, 85.7%), and the median reported age was 39.5 years old (IQR = 13 years old, range, 16–80 years old).

**TABLE 2:** Patients' characteristics.

Total cases (*n* = 84)	(% [*n*])
Age (median [IQR]), years	39.5 (13)
Sex	
Women	85.7 (72)
Men	14.3 (12)
Aetiology of Cushing syndrome	
ACTH dependant	71.4 (60)
Pituitary source	70.2 (59)
Ectopic source	1.2% (1)
ACTH independent	28.6 (24)
Adrenal adenoma	23.8 (20)
Adrenal hyperplasia	4.8 (4)
Treatment of Cushing syndrome^a^	
Surgical resection	97.6 (82)
Medical therapy	19.0 (16)
Radiation therapy	8.3 (7)
Biochemical remission reported	79.8 (67)

^a^ Adds up to more than 100% as multiple reasons could be documented.

The most common aetiology of CS was pituitary adenoma (*n* = 59), followed by adrenal adenoma (*n* = 20) and adrenal hyperplasia (*n* = 4). One patient had a pulmonary neuroendocrine tumour secreting ACTH [8]. All patients but two underwent surgical resection for definitive management of CS. One patient underwent medical management alone with pasireotide [18], and the other had resolution of CS secondary to an adrenal adenoma following adrenal haemorrhage after a motorcycle collision [14]. All patients included in our analysis had documented clinical remission of hypercortisolism, and biochemical remission was reported in 67 cases (79.8%).

**FIGURE 3:**
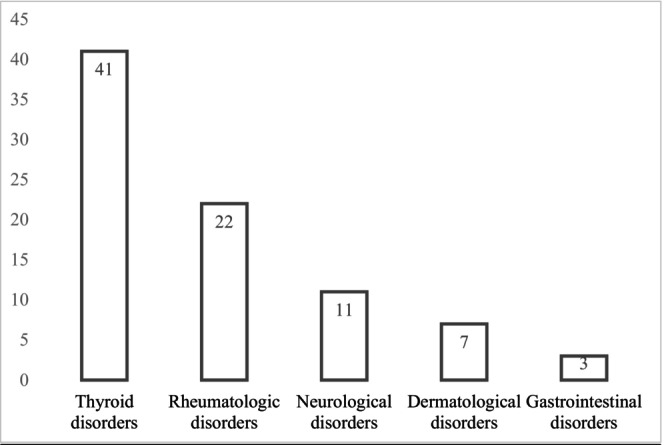
De novo conditions, by system.

The most commonly reported de novo conditions following CS remission were thyroid disorders (*n* = 41, 48.8%), including 34 cases of thyroiditis [9, 10, 11, 17, 18, 19, 20, 21, 22, 23] and seven cases of Graves disease [8, 9, 21, 24, 25, 26]. Rheumatological disorders were the second most commonly reported conditions (*n* = 22, 26.2%) with cases of sarcoidosis (*n* = 13) [8, 14, 27, 28, 29, 30, 31, 32, 33, 34, 35], systemic lupus erythematosus (*n* = 2) [9, 36], rheumatoid arthritis (*n* = 2) [37, 38], seronegative arthritis (*n* = 2) [37, 39], polymyalgia rheumatica (*n* = 1) [40], giant cell arteritis (*n* = 1) [9] and retinal vasculitis (*n* = 1) [39] (see Figure 3 and Table 3). Further characterisation of thyroid disorders and sarcoidosis is detailed below.

**TABLE 3:** Characteristics of de novo conditions.

De novo conditions, by system (*n* = 84)	(% [*n*])
Thyroid disorder	48.8 (41)
Silent thyroiditis	23.8 (20)
Hashimoto thyroiditis	13.1 (11)
Graves disease	8.3 (7)
De Quervain thyroiditis	3.6 (3)
Rheumatologic disorder	26.2 (22)
Sarcoidosis	15.5 (13)
Systemic lupus erythematous	2.4 (2)
Rheumatoid arthritis	2.4 (2)
Seronegative arthritis	2.4 (2)
Polymyalgia rheumatica	1.2 (1)
Giant cell arteritis	1.2 (1)
Retinal vasculitis	1.2 (1)
Neurological disorder	13.1 (11)
Idiopathic intracranial hypertension	6.0 (5)
Multiple sclerosis	2.4 (2)
Lymphocytic hypophysitis	2.4 (2)
Myasthenia gravis	1.2 (1)
Acute disseminated encephalitis	1.2 (1)
Dermatological disorder	8.3 (7)
Psoriasis	3.6 (3)
Rash	3.6 (3)
Generalised rash	1.2 (1)
Rosacea‐like rash	1.2 (1)
Eczematous rash	1.2 (1)
Angioedema	1.2 (1)
Gastrointestinal disorder	3.6 (3)
Celiac disease	1.2 (1)
Primary biliary cirrhosis	1.2 (1)
Sclerosing pancreatocholangitis	1.2 (1)

We identified 11 cases of neurological disorders, including idiopathic intracranial hypertension (IIH) (*n* = 5) [15, 16, 41, 42, 43], multiple sclerosis (*n* = 2) [44, 45], lymphocytic hypophysitis (*n* = 2) [46, 47], acute disseminated encephalomyelitis (*n* = 1) [48] and myasthenia gravis (*n* = 1) [49]. IIH has been associated with both primary adrenal insufficiency and steroid withdrawal [15]. Glucocorticoids are not routinely used as first‐line treatment of IIH (due to the risk of rebound intracranial hypertension upon withdrawal); however, three of the five cases included in this review were successfully treated with higher doses of steroids [15, 16, 41]. Given this association, IIH was considered a steroid‐responsive condition for the purpose of this review. Acute disseminated encephalomyelitis is a rare autoimmune disease, causing widespread inflammation of the brain and spinal cord, often associated with preceding viral infection or vaccination. However, as first‐line treatment for this condition is high dose corticosteroids, we considered it a steroid‐responsive condition and was included in this review [50].

Seven dermatological cases were identified in our scoping review including psoriasis (*n* = 3) [8, 9], rash (*n* = 3) [8] and angioedema (*n* = 1) [51].

Gastrointestinal conditions were the least reported (*n* = 3) with one case of celiac disease [52], one case of primary biliary cirrhosis [8] and one case of sclerosing pancreatocholangitis [53].

The median reported time between the treatment of CS and the onset of symptoms of de novo condition was 4.1 months (IQR = 3.75 months, range, 10 days to 27 years). Most patients (*n* = 50, 59.5%) were receiving corticosteroids at the time of onset. Only 22 cases (26.2%) explicitly reported a timeline from discontinuation (*n* = 6) or tapering (*n* = 16) of corticosteroid dose to the onset of symptoms, with a median time of 1.75 months (IQR = 3 months, range 7 days‐7 months). Thirty‐nine patients (46.4%) were subsequently treated with corticosteroids (either re‐initiated or at an increased dose). Remission or clinical stability of the de novo condition was reported in 66 cases (78.6%), while seven cases (8.3%) remained uncontrolled, and in 11 cases (13.1%), the outcome was not reported. Of the 44 cases where time to remission was reported, the median time was 3 months (IQR = 4.2 months, range 1–24 months).

### 4.1 Thyroid Disorder Cases

Amongst the seven cases of Graves disease, six patients were women and the median age at onset was 44 years old (IQR = 10.5 years old, range, 33–58 years old). Four patients had a pituitary adenoma, two had an adrenal adenoma and one had unilateral adrenal hyperplasia. They all presented with classical signs and symptoms of this condition such as weight loss, tachycardia, goitre and/or orbitopathy. The median time to onset was 5 months (IQR = 3.55 months, range 2–27 months). The majority (5/7) were not on steroids at the onset of Graves disease, and six required additional treatment with antithyroid medications.

Of the 34 cases of thyroiditis, 30 patients were women and the median age at onset was 35.5 years old (IQR = 15.5 years old, range 16–80 years old). Twenty‐three patients had a pituitary adenoma, eight had an adrenal nodule and three had adrenal hyperplasia. Twenty patients presented with silent thyroiditis, 11 patients presented with Hashimoto thyroiditis and three patients presented with De Quervain (subacute) thyroiditis with fever, neck pain and malaise. Time to onset ranged from 1 to 9 months, with a median of 4.85 months (IQR = 3 months). Twenty‐three patients were on steroids at the time of onset, and all patients with De Quervain thyroiditis (*n* = 3) and most patients with transient thyrotoxicosis (*n* = 13) were managed with increased corticosteroid doses.

### 4.2 Sarcoidosis Cases

Amongst the 13 identified sarcoidosis cases, 10 patients were women and the median age at onset was 41 years old (IQR = 9, range 27–45 years old). Eight patients had Cushing disease while five had an adrenal adenoma, and all had undergone surgical resection, except for the patient with adrenal haemorrhage. The time between CS remission and onset ranged from 2 weeks to 17 months, with a median time of 3 months (IQR = 3). Twelve patients had skin manifestations with either painless subcutaneous nodules or erythema nodosum, while our case did not have any skin manifestations. Twelve patients had pulmonary involvement with bilateral mediastinal and/or hilar lymphadenopathy (*n* = 11) or abnormal pulmonary function test (*n* = 1). Eleven patients were on corticosteroids at the time of onset, of which four required increased doses, while the other seven patients did not require additional steroids. The remaining two patients who were not receiving corticosteroids were started on them for the management of sarcoidosis.

## 5 Discussion

Our scoping review identified 20 conditions following the remission of CS, suggesting that the resolution of glucocorticoid excess and its associated immunosuppressive effect can unmask these diseases. The majority of cases were female, which is in keeping with the epidemiology of Cushing syndrome [2] as well as of autoimmune disease in the general population [54, 55]. Thyroiditis, sarcoidosis and Graves disease were the most commonly reported conditions. The prevalence of de novo thyroid disorders in our review may reflect that autoimmune and inflammatory thyroid diseases are common in the general population [55, 56, 57]. However, detection and publication bias may also play a role, as we presume endocrinologists are more likely to diagnose and report thyroid disorders versus non‐endocrine conditions.

Though most de novo conditions presented within 1 year of CS remission, the reported timing of onset was variable, ranging from 10 days to 27 years. This may reflect differences in post‐remission glucocorticoid doses, weaning schedules and responsiveness of various conditions to glucocorticoids. We emphasise that we cannot prove a causative link between CS remission and the emergence of the de novo condition in our case or the other reported cases. Due to the heterogeneity in glucocorticoid requirements and tapering schedules post‐CS remission [58, 59], as well as our aim characterising this clinical presentation, we chose not to specify the timing of the onset of de novo conditions in our inclusion criteria. However, we suggest that the emergence of a condition further out from the withdrawal of supraphysiologic glucocorticoids is less likely to be related to the previous state of hypercortisolism. We are dubious about one case in particular [46] that reported a patient with the onset of lymphocytic hypophysitis 27 years post subtotal adrenalectomy for CS, despite tapering off glucocorticoids within a month of surgery. The second case of lymphocytic hypophysitis occurred 7 years after the remission of Cushing disease, but there is no mention of whether the patient was still on exogenous glucocorticoids at the time of onset [47]. With the exclusion of these two cases, the onset of de novo conditions ranged from 10 days to 60 months, the latter case [8] being the emergence of psoriasis following the delayed normalisation of hypercortisolism with medical therapy and radiotherapy, which is more clinically plausible.

Our case highlights the challenge of diagnosing a new systemic disorder when features of AI and/or GWS are concurrently present. To avoid diagnostic delay in this setting, we emphasise that clinicians should have a low threshold to investigate symptoms atypical for AI or GWS including (but not limited to) skin changes, neurological symptoms, pulmonary symptoms and symptoms of thyroid disease, particularly if symptoms present or worsen as supraphysiologic glucocorticoids are weaned.

### 5.1 Strengths and Limitations

To our knowledge, this is the first scoping review to synthesise the existing literature on autoimmune, inflammatory and steroid‐responsive conditions following Cushing syndrome remission. We adhered to PRISMA scoping review methodology and developed a comprehensive literature search strategy. However, we limited our review to publications in English and French, which resulted in the exclusion of 17 articles. The reported cases are subject to diagnostic and publication bias; therefore, our review may not encompass all de novo conditions that can present in this setting. As outlined above, we cannot establish a causative link between the remission of CS and the emergence of the reported de novo conditions.

## 6 Conclusion

Our scoping review identified several cases of distinct autoimmune, inflammatory or steroid‐responsive conditions emerging following the remission of Cushing syndrome, amongst which thyroid disorders and sarcoidosis were the most commonly reported. Delineating such conditions from the expected clinical course of GWS and/or AI can be a challenge; therefore, clinicians should have a low threshold to investigate any atypical symptoms following the remission of Cushing syndrome.

## Author Contributions

**Noémie Desgagnés:** Conceptualization (equal); data curation (equal); formal analysis (equal); investigation (equal); methodology (equal); project administration (equal); visualization (equal); writing – original draft (equal). **Laura Senior:** Data curation (equal); formal analysis (equal); investigation (equal); writing – original draft (equal). **Daniel Vis:** Writing – review and editing (equal). **Katayoun Alikhani:** Writing – review and editing (equal). **Kirstie Lithgow:** Conceptualization (equal); data curation (equal); investigation (equal); methodology (equal); project administration (equal); supervision (lead); writing – review and editing (lead).

## Conflicts of Interest

The authors have declared no conflicts of interest.

## Data Availability

The data that support the findings of this study are available from the corresponding author upon reasonable request.

## Appendix 1 MEDLINE search strategy

### 1.1

**Table:**

#	Query	Results
1	exp Cushing Syndrome/	12,803
2	cushing* syndrome.tw,kf.	10,669
3	cushing* disease.tw,kf.	5341
4	1 or 2 or 3	18,042
5	(de novo adj2 steroid*).tw,kf.	160
6	exp Sarcoidosis/	26,609
7	sarcoid*.tw,kf.	29,517
8	exp Polymyalgia Rheumatica/	2725
9	PMR.tw,kf.	3328
10	polymyalgia rheumatica.tw,kf.	2890
11	exp Multiple Sclerosis/	67,479
12	MS.tw,kf.	395,579
13	multiple sclerosis.tw,kf.	87,101
14	exp Autoimmune diseases/	527,961
15	autoimmun*.tw,kf.	199,531
16	exp Systemic lupus erythematosus/	65,187
17	SLE.tw,kf.	38,508
18	systemic lupus erythematosus.tw,kf.	56,501
19	exp Rheumatoid arthritis/	122,521
20	RA.tw,kf.	86,402
21	rheumatoid arthritis.tw,kf.	117,236
22	arthritis*.tw,kf.	201,619
23	exp Sjogren syndrome/	14,144
24	sjogren syndrome.tw,kf.	3325
25	exp celiac disease/	21,506
26	celiac disease.tw,kf.	13,896
27	exp myasthenia gravis/	16,576
28	myasthenia gravis.tw,kf.	16,140
29	exp Crohn disease/	43,066
30	crohn disease.tw,kf.	5001
31	crohn*.tw,kf.	54,352
32	exp Ulcerative colitis/	39,053
33	ulcerative colitis.tw,kf.	46,316
34	UC.tw,kf.	25,420
35	colitis*.tw,kf.	77,427
36	exp dermatitis/	112,602
37	dermatitis.tw,kf.	68,667
38	exp vasculitis/	102,088
39	vasculitis.kw,kf.	6283
40	exp myositis/	21,858
41	exp thyroiditis/	15,345
42	thyroid*.tw,kf.	216,663
43	exp IgG4/	154,671
44	igg4.tw,kf.	10,905
45	exp encephalopathy/	1,361,632
46	encephalopathy.tw,kf.	53,011
47	steroid responsive.tw,kf.	1576
48	5 or 6 or 7 or 8 or 9 or 10 or 11 or 12 or 13 or 14 or 15 or 16 or 17 or 18 or 19 or 20 or 21 or 22 or 23 or 24 or 25 or 26 or 27 or 28 or 29 or 30 or 31 or 32 or 33 or 34 or 35 or 36 or 37 or 38 or 39 or 40 or 41 or 42 or 43 or 44 or 45 or 46 or 47	3,122,540
49	4 and 48	5865
50	exp Case Reports/	2,289,770
51	case report*.tw,kf.	466,902
52	exp Observational Study/	132,022
53	observational stud*.tw,kf.	147,088
54	case series.tw,kf.	96,054
55	50 or 51 or 52 or 53 or 54	2,676,537
56	4 and 48 and 55	1479

Ovid MEDLINE(R) and Epub Ahead of Print, In‐Process, In‐Data‐Review & Other Non‐Indexed Citations and Daily <1946 to September 08, 2022>.
